# Prevalence and Determinants of Khat (*Catha edulis*) Chewing among High School Students in Eastern Ethiopia: A Cross-Sectional Study

**DOI:** 10.1371/journal.pone.0033946

**Published:** 2012-03-30

**Authors:** Ayalu A. Reda, Asmamaw Moges, Sibhatu Biadgilign, Berhanu Y. Wondmagegn

**Affiliations:** 1 Department of Public Health, College of Health Sciences, Haramaya University, Harar, Ethiopia; 2 School of Public Health, College of Health Sciences, Addis Ababa University, Addis Ababa, Ethiopia; 3 Department of Epidemiology and Biostatistics, College of Medical and Health Sciences, Jimma University, Jimma, Ethiopia; 4 Department of Environmental Health Science, College of Health Sciences, Haramaya University, Harar, Ethiopia; University of Granada, Spain

## Abstract

**Background:**

Use of psychoactive drugs such as khat leaves (*Catha edulis*) alter moods and emotional state and lead to adverse effects on the health and social life of users. Ethiopia is a major producer and exporter of khat in east Africa and the majority of the khat comes from the eastern part of the country, however, no studies have been conducted to investigate the habit in this area. This study was conducted to assess the prevalence and predictors of khat chewing among high school students in Harar, eastern Ethiopia.

**Methodology:**

The study was conducted among 1,890 secondary school students in Harar town in April 2010. A structured self-administered questionnaire was used for data collection. Descriptive statistics and logistic regression were performed to examine the prevalence and predictors of khat chewing.

**Result:**

The overall prevalence of khat chewing among the sample was 24.2% (95% CI 22.2%–26.2%). About 28.5% of females and 71.5% of males had chewed khat. Older age (OR 1.31; 95% CI 1.16–1.49), male gender (OR 2.10; 95% CI 1.50–2.93), Muslim religion (OR 1.88; 95% CI 1.17–3.04), having friends who chewed khat (OR 7.93; 95% CI 5.40–11.64), and availability of someone with a similar habit in the family (OR 1.50; 95% CI 1.07–2.11) were found to be independent predictors of chewing.

**Conclusion:**

A significant proportion of students chew khat. The use of khat is significantly associated with age, gender, Muslim religion, peer influence and habit of family and other relatives among students. Measures such as educational campaigns need to be instituted to create awareness among school adolescents and their parents in order to reduce the prevalence of the habit and its adverse social and health consequences.

## Introduction

Khat (*Catha edulis*) is an evergreen plant that is extensively cultivated in the highlands of Ethiopia [1] and surrounding countries like Kenya and Yemen [2]. It is a bushy plant whose leaves are the source of a naturally occurring amphetamine-like substance. It is highly abused for its stimulant properties [3]. Stimulation is commonly obtained by chewing the leaves privately or in small social gatherings. Its effects on the chewer include increased levels of energy, increased self-esteem, euphoria, increased libido, excitement, and increased proclivity for social interaction [2], [4], [5]. The practice of khat chewing dates back thousands of years in eastern Africa and the Arabian Peninsula where the plant is widely cultivated and known by a variety of names like chat or khat in Ethiopia, and qaat or jaad in Somalia [6].

In Ethiopia, khat is commonly used for stimulation and social recreation. A significant number of students consume khat to be alert and wakeful at night especially during examination periods 7,8,9,10. In a similar manner other sections of the community like teachers and farmers chew khat in order to reduce fatigue and increase performance [7].

Despite its popularity, there is ample evidence of the adverse effects of habitual use of khat on mental, physical and social well-being [4], [11]. Mentally and physically, it leads to problems like depression and anxiety, psychosis, impotence, cardiovascular events, liver failure and stomach ulcers among others [7], [12]. Socially, khat chewing results in diversion of household income among poor families and reduced productivity [7], [13]. It has also been implicated with use of drugs, smoking, alcohol and sexual risk behaviors [14], [15], [16].

Even though khat is popular among youth and adults in Ethiopia, there are only a few studies that have investigated its prevalence in different sections of the community. Particularly in eastern Ethiopia where the majority of khat in the country is produced and exported abroad, no studies have been conducted to investigate the habit. So we desire to inform decision and policy makers in their efforts to understand and reduce khat use. The aim of this study is therefore to assess khat chewing among school adolescents in Harar town, eastern Ethiopia.

## Methods

### Ethics statement

Ethical clearance was obtained from the Institutional Research Ethics Review Committee of Haramaya University, College of Health Sciences. A letter of cooperation was written from Haramaya University and further approval was obtained from the regional health bureau and the surveyed schools. The objective of the study was explained to the study participants. The students were briefed about the confidentiality of their response and the importance of providing correct and accurate information, and that participation was voluntary. All participants included in the study have provided a written consent. We did not believe that the questionnaire provided to students compromises ethical principles. As a result parental approval was not sought; the Ethical Review Board has approved this decision.

### Study setting

The study was conducted in Harar town located 525 Km east of Addis Ababa, the capital city of Ethiopia. Harari Regional State is one of the nine regions in Ethiopia and Harar town is its capital city. The town has nine high schools from grades 9 to 12. The total number of students enrolled in these schools in the academic year at the time of this study was 6,523.

### Study design

A descriptive cross-sectional study was conducted in April 2010 among high school students to assess the prevalence of khat chewing and its determinants. All schools were included in the study and proportional stratified sampling technique was employed to obtain study units - where a proportional sample of students were selected from each school and grades based on their enrolment size at the time of the study. Within grades, all students in the selected classes participated regardless of their age. A total of 1,890 students, representing 29% of the study population were surveyed using this procedure.

### Data collection procedure

A self-administered structured questionnaire consisting of open and closed ended questions was developed from a review of the literature. The anonymous questionnaire covers socio-demographic factors in addition to khat chewing habits. The English version of the questionnaire was translated into Amharic, the official language of the study area, by a panel of experts fluent in the language. It was then translated back in to English by another person to ensure consistency with the English language questionnaire. The Amharic language questionnaire was used to collect data. It was employed after pre-testing on 95 students in schools outside but close to the study area and corrections was made thereafter to improve the clarity of some items. The participants anonymously responded to the items on the questionnaire using pen or pencils. The questionnaire is attached as a supporting information in File S1 below.

### Operational definition

A student was considered a chewer if s/he respond yes to the question ‘Have you chewed khat previously?’ Then follow up questions were employed to collect information such as place and frequency of chewing.

### Statistical analysis

The data were coded, entered, cleaned, and analyzed using IBM® SPSS® Statistics, version 15 for Windows. Descriptive statistics were conducted using frequencies and proportions. Bivariate and multivariate analyses were carried out using logistic regression to examine the relationship between the outcome variable of khat chewing and selected determinant factors. The multivariate logistic regression model was derived through simultaneous entry analysis; model diagnostics such as outliers and multicollinearity were examined through linear regression. Adjusted and unadjusted odds ratios (OR) and their 95% confidence intervals (CI) were used as indicators of strength of association. A p-value of 0.05 or less was used as the cut-off level for statistical significance.

## Results

### Socio-demographic characteristics of respondents

Out of a total of 1,890 students provided with questionnaires, 1,721 completed and returned them, providing a response rate of 91.1%. Of these 856 (50.1%) were males and 851(49.9%) females. The mean (SD) age of the respondents was 16.4 (1.60) years (see Table 1).

**Table 1:** Socio-demographic characteristics of the sampled (n = 1,721) high school students in eastern Ethiopia.

Variables	Frequency	Percent
Sex		
Female	851	49.9
Male	856	50.1
Age		
15–19	1085	63.5
20–25	622	36.4
Religion		
Orthodox	901	52.8
Protestant	36	2.1
Catholic	549	32.2
Muslim	191	11.2
Others*	29	1.7
Grade		
9^th^	839	48.9
10^th^	394	23.0
11^th^	253	14.7
12^th^	230	13.4
Living with		
Parents	1288	92.5
Friends	27	1.9
Relatives	43	3.1
Alone	34	2.4

* Other faiths include traditional, Jehovah Witness.

### Khat chewing practice

A total of 427 (24.2%; 95% CI 22.2%–26.2%) students ever chewed khat. Of the chewers, 89 (20.9%) chewed khat daily and 128 (29.9%) used Shisha when they chewed khat. The mean (SD) age of chewing debut was 15.1 (2.33) years. Of these, the majority was males (305, 71.5%) and 171 (42.0%) belonged to grade 9. Out of those who chewed khat, 144 (33.6%) spent more than 26 birr (mean = 26.4 birr) per week (amounting to $ 2.03; exchange rate at the time of the study, 1 USD = 14 Ethiopian Birr). Seventy-eight percent got the money from their family and 22% from their friends. In addition to khat chewing, 186 (43.5%) and 142 (33.3%) students drank alcohol and smoked cigarettes respectively (please refer to supporting file, File S2, for table).

### Factors independently associated with khat chewing

The multivariate logistic regression analysis showed that the odds of chewing were eight times higher with students who had friends who chewed khat compared to those who didn't (OR 7.93; 95% CI 5.40–11.64). Male students had two times higher odds of chewing khat compared to female students (OR 2.10; 95% CI 1.50–2.93). Those students who are living with khat chewers had a 1.5 times higher odds of chewing compared to those who did not (OR 1.50; 95% CI 1.07–2.11). As the age of the students increased by one year the odds of khat chewing increased by 1.31 (OR 1.31; 95% CI 1.16–1.49). Muslim students had close to two times higher odds of chewing khat compared to Orthodox Christians (OR 1.88; 95% CI 1.17–3.04). Grade level of students was statistically significant in the binary (crude) analysis but not when adjusted for other variables in the final model (Table 2).

**Table 2:** Logistic regression model estimates of risk factors for chewing khat among high school students in eastern Ethiopia (n = 1,721)§.

Explanatory variable	OR (95% CI)	P-value	Adjusted odds ratio (95% CI)	P-value
Sex				
Male	3.29(2.58–4.19)	<0.001	2.10 (1.50–2.93)	<0.001
Female	1.00		1.00	
Age	1.44 (1.33–1.55)	<0.001	1.31 (1.16–1.49)	<0.001
Grade				
9^th^	1.00		1.00	
10^th^	0.93 (0.70–1.25)	0.642	1.02 (0.73–1.43)	0.893
11^th^	1.04 (0.741–1.45)	0.831	0.99 (0.67–1.45)	0.954
12^th^	0.45 (0.328–0.61)	<0.001	0.58 (0.41–1.83)	0.333
Religion				
Orthodox Christian	1.00		1.00	
Muslim	1.95 (1.26–3.00)	0.003	1.88 (1.17–3.04)	0.010
Protestant	0.38 (0.193–0.73)	0.004	0.38 (0.17–0.84)	0.017
Catholic	0.74 (0.59–0.94)	0.015	0.71 (0.49–1.03)	0.070
Others	1.27 (0.514–3.16)	0.601	1.43 (0.50–3.99)	0.511
Have friends who chewed khat				
Yes	12.46 (9.19–16.89)	0.001	7.93 (5.40–11.64)	<0.001
No	1.0		1.0	
Living with khat chewers				
Yes	2.94 (2.33–3.78)	0.024	1.50 (1.07–2.11)	0.02
No	1.0		1.0	

§ No multicollinearity was detected in the model, the mean variance inflation factor (VIF) was 2.05 and no variable had a VIF of more than 10. On case-wise diagnostics there were no residuals with values more than 3 standard deviations, indicating that there are no outlier cases.

## Discussion

The findings of this study reveal the self-reported prevalence of khat chewing among the study population to be 24.2% (95% CI 22.2%–26.2%). The independent predictors of khat chewing were age, gender, Muslim religion, and existence of chewing habit among friends, family and relatives.

Social desirability bias is a potential limitation of this study. However, we believe that this risk is minimized because we employed a self-administered tool to gather data and provided a good level of privacy during data collection. Furthermore, because of the underdeveloped nature of khat research, we were unable to use standardized questionnaires and/or operational definitions to assess the magnitude of khat chewing in a comparable manner. However, we derived the items of the questionnaire from literature review in order to solve this problem and enable comparability of the findings. We also acknowledge that the findings of this study may not be necessarily applicable to out-of-school youth or adolescents where the use of khat is expected to be higher. We obtained a good level of response (91.1%). However, the respondents may not necessarily represent all students since those absent at the time of the data collection may be delinquents who are expected to have a higher proportion of chewing.

Generally, the prevalence of khat chewing in this study seems to be lower than studies carried out in other parts of the country. A study from south-western Ethiopia [17] revealed that the prevalence of khat chewing among secondary school students to be 64.9%. A survey carried out in a rural community in southern Ethiopia found a prevalence of 50% [1], where as a survey of 1,200 adults from another rural Ethiopian community reported prevalence of 31.7% [11]. These figures are higher than what we found. This may be because of the fact that most of these studies included samples of students who come from rural communities who are directly economically dependent on khat through cultivation and marketing it. This may mean that they are also likely to develop the habit of consuming khat. Our sample included students from both semi-urban and urban backgrounds and whose parents include not only farmers but also civil servants and traders. In neighboring Somalia about 36.4% of respondents reported to have chewed khat in the week preceding the interview [18]. The Somali sample is composed of combatants reportedly under severe stress and in a context of social disruption which potentially increases substance use, hence the difference with our study. Our finding is similar to a report from Jazan region of Saudi Arabia in which the prevalence of khat use among high school students was 21.4% [19]. The study may have shown similarity with the findings of the study since Jazan region is located at 70 kilometers near the border with Yemen, which is a major khat producing country [19]. However, this should be interpreted carefully since the similarity could also be due to chance.

Furthermore, the possible explanations for the observed differences in khat chewing could be due to differences in sample characteristics, in the definitions used by studies, cultural differences in understanding of the amount of chewing and methodological differences. For instance, as in alcohol research [20] measures of khat chewing could be frequency (e.g. daily, past week, past month etc. [8], [15], [16], [18]) or amount based which seems to be less common (e.g. bundles, money spent, etc. [18]). However, these are still not well refined and standardized currently, the commonest measure of khat chewing used by studies seem to be based on categorical yes or no response questions with some kind of time indicator. In turn, the time variable creates problems of comparability since there are no standardized frames of reference.

In this study the mean (SD) age for starting chewing khat was 15.1 (2.33) years. This figure was reported to be 17.3 (3.02) years among college students in a study from northwestern Ethiopia [21]. This shows that the participants in our study area start the habit early. This could be explained by the fact that the cultivation and consumption of khat is practiced widely in the eastern Ethiopia and it is more a part of the culture of the study area than that of the northern regions. Furthermore it is known that in predominantly khat using cultures children and adolescents may be encouraged to ‘try’ khat chewing by parents and other members of the community. Apart from similar observations by the authors of this report, a study from Yemen also reports that school-aged children as young as eight years are provided with khat with the belief that they could study better and become energetic [2].

Muslims (OR 1.88; 95% CI 1.17–3.04) and male (OR 2.10; 95% CI 1.50–2.93) students were found to have higher odds of chewing khat in this study. This is similar to a study conducted in Gondar [22] and Butajira [1] among high school students. Male college students in Gondar had higher odds (OR 3.69; 95% CI 2.10–6.26) of khat chewing than female students [21]. A similar finding has been reported among secondary school students in Saudi Arabia showing significant differences in chewing between males and females [19]. This might be due to the common tendency of males to abuse substances compared to females and to the greater cultural acceptance of male substance use in Ethiopia and among other khat consuming countries. Even though it is not sanctioned by the religion, it is the authors' experience that in the study area, as opposed to Christians, Muslims commonly report using khat to stay awake for prayers. In this study, even though the students may not necessarily use it for prayers, being born in a family that uses it for prayer may influence its use. Our findings seem to also agree with previous reports from Ethiopia [1], [23].

While own and friends' homes seem to be the most common (90%) place where chewing ceremony takes place, about 6.7% students reported that they used special rooms for khat chewing. Commonly in Ethiopia as well as countries such as Yemen, both commercial and non-commercial khat chewing rooms are available [2]. In our study area these rooms have radios, music players, TV and floor mats or mattresses. The rooms are clean and not used for other purposes but may be used individually or in groups by reclining or sitting on the floor. A lesser number of students reported to use the rooms. This may be because these rooms are commonly reserved for adults or that they may not afford the expenses of a commercial khat chewing room.

The subjective reasons given for khat chewing in this study were “to get concentration”, “peer pressure” and “for enjoyment” among others. Reasons given by college students in north west Ethiopia for starting cigarette smoking and khat chewing were 119 (40.5%) to keep alert while reading and 97 (33.0%) for relaxation with friends [21]. The main reasons mentioned for starting chewing were “peer pressure” and “for relieving stress” [19]. Other reasons for chewing khat reported in the literature include for religious prayer, to pass time, and to accompany or socialize with family members [16].

In this study about 43.5% and 33.3% students drank alcohol and smoked cigarettes respectively in addition to khat chewing. This indicates that there is clustering of substance use behaviors among students with khat chewing habit [10], [24], [25]. Even though it may be difficult to generalize to other contexts, in our study area khat is an entry point for cigarette smoking and alcohol use. Alcohol and cigarettes are commonly used to break the depression that ensues once the stimulation from khat has subsided. Once chewers spent the afternoon chewing, in the evening they commonly go out to drink alcohol in bars. Furthermore, the fact that a student is already using khat may create a liberal view about other substances or may expose him or her to people who have such beliefs as discussed below. In a similar manner, a report among Somali khat chewers in London indicated that 60 percent of chewers also smoked [26]. This may imply that efforts at tackling khat chewing might also be productive in reducing multi-substance use or that efforts at tackling other substances need to also consider tackling khat use.

In this study the habit of chewing khat by friends, parents and other family members was associated with an increased odds of chewing khat among the study participants. This finding is in agreement with the findings among Gondar high-school students [27], youth from Butajira in southern Ethiopia [22] and Kenya [28]. In other studies of substance use it is also demonstrated that friends and parents have a significant influence on adolescents. For instance, substance use was higher among youth who perceived peers or family members to be using drugs and was inversely correlated with increased parental monitoring and supervision [29] and results indicate the more the numbers of parents and friends using substances, the higher the risk of adolescent substance use, as were more substance offers. Poorly monitored adolescents are more likely to use drugs, and drug-using adolescents seek out like-minded friends. Once an adolescent establishes an association with drug using peers, her or his own substance use approaches their level and habit of use [30]. This might be due to the influence of social environment and that young people tend to imitate and practice whatever they observe from their friends, elders and parents. This indicates that friends and parents should be an integral part of adolescent substance use risk reduction approaches and strategies. However, non-using parents had a buffering effect on friends' influences to use substances, such that friends' use did not influence adolescent use when parents were non-users, and the effects of substance offers on refusal were weaker [31].

In conclusion, khat chewing is prevalent among high school students in the study area. Khat chewing is significantly associated with male gender, peer influence and similar habit among family members, and being a Muslim. This finding indicates that there is a need for early intervention that targets pre-secondary and high school students to reduce the health, financial and social consequences of khat use. This may involve measures to create awareness among students about its consequences. Parental substance use should also be addressed in adolescent substance use prevention programs.

## Supporting Information

File S1 Contains the questionnaire used for data collection.
(DOC)

File S2 Contains table of characteristics of chewers.
(DOC)
