# Supplementary material for: Prevalence and Determinants of Khat (Catha edulis) Chewing among High School Students in Eastern Ethiopia: A Cross-Sectional Study
Source: PLoS One. 2012 Mar 30;7(3):e33946. doi: 10.1371/journal.pone.0033946 (PMC3316517; doi:10.1371/journal.pone.0033946)
Supplement: File S1 — Contains the questionnaire used for data collection. (DOC) [file pone.0033946.s001.doc]

Questionnaire to assess khat chewing

**Please encircle on the item containing your response and if you change your answer, erase your old answer completely, and mark your new choice clearly. Do not write you name or ID.**

The first eight questions inquire about your socio-demographic characteristics

**1. How old are you? Specify…………..**

**2. What is your sex?**

1. Female □
2. Male □

**3. In what grade are you?**

1. 9th grade □
2. 10th grade □
3. 11th grade □
4. 12th grade □

**4. What is your ethnicity?**

1. Amhara □
2. Oromo □
3. Somali □
4. Tigre □
5. Gurage □
6. Adere □
7. others □

**5. What is your religion?**

A. Orthodox □

B. Muslim □

C. Protestant □

D.Catholic □

E. Others □

**6. You are currently living with __________________________________**

**7. Your marital status is**

A. Single □

B. Married □

C. Divorced □

D. Widowed □

**The next 11 questions are asking about habit of khat chewing**

**8. Have you ever chewed khat?**

A. yes □

B. No □

**9. If your answer for the above question is yes, how old were you when you started to chew khat?_____________________________**

**10. Where do you usually chewing khat**?

A. I have never chewed khat □

B. If you do, specify______________________________

**11. During the past 30 days, how many days did you chewed khat?**

A. I have never chewed khat □

B. If you did, specify __________________________

**12. How many birr do you usually spend for khat per week?**

A.I have never chewed khat □

B. If you do, specify in birr_______________________

**13. From where do you get the money?**

A. I have never chewed khat □

B. If you chewed, specify your source________________________

**14. With whom do you usually chew khat**?

A. I have never chewed khat □

B. With my friends □

C. With my family □

**15. Do you use shisha when you are chewing khat?**

**A.** Yes □

B. No □

**16. How did you usually get the khat when you are interested to chew khat?**

A. I have never chew khat □

B. From a school friend □

C. From khat store □

D. From my family □

**17. Individuals living with you chew khat?**

A. Yes □

B. No □

**18. If your answer to question 17 is yes, what is their relationship with you?_____________________________**

**19. During the past 12 months, did you ever try** *to quit* **chewing khat?**

A. I did not chew during the past 12 months

B. Yes □

C. No □

**20. Have you ever tried cigarette smoking, even one or two puffs?**

B. Yes □

C. No □

**21. during your life, have you ever drunk alcohol?**

B. Yes □

C. No □

Amharic version

**mGlÅÝ yÅT m”MN y¸Ã-Â m-YQ**

- MRÅHN ¼>N¼ xKBB¼b!
- MRÅ>N ¼>N¼ kqyRK¼>¼ ymjm¶ÃWN MRÅ x_Í ¼ð¼

ymjm¶Ããc$ 8 _Ãq&ãC ¥Hb‰êE Ñ<ÇÄ‹” ÃSKŸK<

1. SNT ›mTH¼>¼ nW) ¼y=rSkW¼>WN XD» ÉF ­___________________­­
2. ò¬

h. wND □

l. s@T □

1. SNt¾ KFL nH¼>¼)

h. z-n¾ □

l. xSr¾ □

/. xS‰xNd¾ □

m. xS‰ h#lt¾ □

1. B/@R

h. x¥‰ □

l. åéä □

/. î¥l@ □

m. TGÊ □

\. g#‰g@ □

r. l@§ □

1. hY¥ñT

h. åRèìKS □

l. ÑSl!M □

/. Pét&S¬NT □

m. µèl!K □

\. l@§ □

6. yMTñ¶W¼rW¼ k¥N UR nW) ___________________

**7. የጋብቻ ሁኔታ**

**ሀ. ያገባ □**

**ለ. ያላገባ □**

y¸ktl#T 11 _Ãq&ãC ÅTN Ymlk¬l#

1. ÅT QmH¼>¼ ¬W”lH¼qEÃl>¼

h. xW”lh# □

l. x§WQM □

1. l_Ãq& 30 mLSH¼>¼ xã kçn m”M STjMR¼¶¼ :D»H¼>¼ SNT nbR)

________________________

1. ÅT yMTQmW¼¸W¼ lMNDN nW)

_________________________________

1. xB²¾WN g!z@ ÅT yMTQmW¼¸W¼ yT nW)

h. Q» x§WQM □

l. yMTQM kçn yT ný yMTQmý) ________________

1. lÅT búMNT MN ÃHL gNzB ¬wÈlH¼Ål>

h. xLQMM □

l. yMTQM kçn yT yBR m-n#N _qS ________________

1. xB²¾WN g!z@ ÅT yMTQmW k¥N UR nW

h. ÅT Q» x§WQM

l. kÙd®c& UR □

/. kb@tsïc& UR □

m. BÒüN □

1. §lûT s§ú qÂT MN ÃHL qÂT ÅT QmhL¼šL

h. ÅT Q» x§WQM □

l. ÅT yMTQM kçn MN ÃHL qN Qm¦L______________________

1. lÅT m”¸Ã y¸çnWN gNzB yM¬gßW¼ß!W¼ kyT nW

___________________

1. ÅT STQM ¹!šH T-q¥lH¼¸Ãl>¼

h. Q» x§WQM □

l. xã □

/. xL-qMM □

1. xBrWH¼>¼ k¸ñ„ sãC mµkL ÅT y¸QM xl

h. xl □

l. ylM □

1. l_Ãq& q$_R 34 mLSH¼>¼ xã kçn y¸QÑT sãC §Nt¼cE¼ MNH¼>¼ ÂcW

________________________

1. §lûT xS‰ h#lT w‰T ÅT §lm”M äKrH ¬W”lH

h. xã □

l. xLäkRk#M □
