# Supplementary material for: Prevalence and Determinants of Khat (Catha edulis) Chewing among High School Students in Eastern Ethiopia: A Cross-Sectional Study
Source: PLoS One. 2012 Mar 30;7(3):e33946. doi: 10.1371/journal.pone.0033946 (PMC3316517; doi:10.1371/journal.pone.0033946)
Supplement: File S2 — Contains table of characteristics of chewers. (DOC) [file pone.0033946.s002.doc]

Table. Characteristics of khat chewer high school students in eastern Ethiopia (n = 427)

| Characteristics of chewers | Frequency | Percent^¥^ |
| --- | --- | --- |
| Sex |  |  |
| Male | 288 | 71.5 |
| Female | 115 | 28.5 |
| Age |  |  |
| 15-19 | 366 | 85.8 |
| 20-25 | 61 | 14.2 |
| Grade |  |  |
| 9^th^ | 171 | 42.0 |
| 10^th^ | 89 | 21.9 |
| 11^th^ | 56 | 13.8 |
| 12^th^ | 91 | 22.4 |
| Religion |  |  |
| Orthodox Christian | 209 | 48.9 |
| Protestant | 16 | 3.7 |
| Catholic | 153 | 35.8 |
| Muslim | 24 | 5.6 |
| Others | 5 | 1.2 |
| Have friends who chewed khat |  |  |
| Yes | 345 | 85.8 |
| No | 57 | 14.2 |
| Living with khat chewers^*^ |  |  |
| Yes | 283 | 70.6 |
| No | 118 | 29.4 |
| Place of chewing |  |  |
| Home | 226 | 52.8 |
| Friends’ home | 159 | 37.2 |
| Special room for khat chewing^§^ | 29 | 6.7 |
| Chewed with: |  |  |
| Friends | 344 | 80.5 |
| Alone | 58 | 13.7 |
| Parents | 25 | 5.8 |
| Main reason for chewing |  |  |
| To concentrate for study or work | 209 | 48.9 |
| For enjoyment, stimulation | 155 | 36.3 |
| Other reasons (bonding, friendship) | 63 | 14.7 |
| **Chewed and drank^¶^** | **186** | **43.5** |
| **Chewed and smoked^¶^** | **142** | **33.3** |
| **Used Shisha with khat^ð^** | **128** | **29.9** |
| **Chew khat daily** | **89** | **20.9** |
| **Has tried to stop chewing** | 240 | 56.2 |

**^*^**Living with people who chew khat include, father, mother, siblings and extended family members. ^§^It could be commercial or a regular room in a home primarily used for khat chewing purposes. ^¥^Percentages calculated based on valid responses to each variable. ^¶^Reference is not made to the timing of the habits (they could take place simultaneously or sequentially). ^ð^Refers to simultaneous use.
